# Correlation of CT-based radiomics analysis with pathological cellular infiltration in fibrosing interstitial lung diseases

**DOI:** 10.1007/s11604-024-01607-2

**Published:** 2024-06-18

**Authors:** Akira Haga, Tae Iwasawa, Toshihiro Misumi, Koji Okudela, Tsuneyuki Oda, Hideya Kitamura, Tomoki Saka, Shoichiro Matsushita, Tomohisa Baba, Yayoi Natsume-Kitatani, Daisuke Utsunomiya, Takashi Ogura

**Affiliations:** 1 Dept. of Radiology, Kanagawa Cardiovascular & Respiratory Center, Yokohama, Japan; 2 Department of Data Science, National Cancer Center Hospital East, Kashiwa, Japan; 3 Department of Pathology, Saitama Medical University, Moroyama, Japan; 4 Dept. of Pathology, Kanagawa Cardiovascular & Respiratory Center, Yokohama, Japan; 5 Dept. of Respiratory Medicine, Kanagawa Cardiovascular & Respiratory Center, Yokohama, Japan; 6 Tokyo Denki University, Tokyo, Japan; 7 Dept. of Radiology, Yokohama City Univ. School of Medicine, Yokohama, Japan; 8 Artificial Intelligence Center for Health and Biomedical Research, National Institutes of Biomedical Innovation, Health and Nutrition, Osaka, Japan; 9 Institute of Advanced Medical Sciences, Tokushima University, Tokushima, Japan

**Keywords:** Lung, Interstitial lung disease, CT, Radiomics

## Abstract

**Purpose:**

We aimed to identify computed tomography (CT) radiomics features that are associated with cellular infiltration and construct CT radiomics models predictive of cellular infiltration in patients with fibrotic ILD.

**Materials and methods:**

CT images of patients with ILD who underwent surgical lung biopsy (SLB) were analyzed. Radiomics features were extracted using artificial intelligence-based software and PyRadiomics. We constructed a model predicting cell counts in histological specimens, and another model predicting two classifications of higher or lower cellularity. We tested these models using external validation.

**Results:**

Overall, 100 patients (mean age: 62 ± 8.9 [standard deviation] years; 61 men) were included. The CT radiomics model used to predict cell count in 140 histological specimens predicted the actual cell count in 59 external validation specimens (root-mean-square error: 0.797). The two-classification model’s accuracy was 70% and the F1 score was 0.73 in the external validation dataset including 30 patients.

**Conclusion:**

The CT radiomics-based model developed in this study provided useful information regarding the cellular infiltration in the ILD with good correlation with SLB specimens.

**Supplementary Information:**

The online version contains supplementary material available at 10.1007/s11604-024-01607-2.

## Introduction

Chronic fibrosing interstitial lung diseases (ILDs) encompass a large and heterogeneous group of parenchymal lung disorders that may be related to either systemic diseases, environmental exposures, or have no known cause. Idiopathic pulmonary fibrosis (IPF) is a common form of chronic, progressive fibrosing ILD that requires treatment with anti-fibrotic drugs [1]. In secondary fibrosing ILDs, such as connective tissue disease-associated ILD and fibrotic hypersensitivity pneumonitis (HP), initial inflammation progresses to fibrosis [2]. Extensive inflammatory cell infiltration is an important histological feature suggestive of secondary ILDs, including connective tissue disease (CTD). Moreover, inflammatory cell infiltration is also part of the hallmark histologic findings of interstitial pneumonia with autoimmune features (IPAF) [3, 4]. In these secondary fibrosing ILDs, immunomodulatory medications play an essential role in the treatment strategy [5–8]. However, fibrotic ILDs frequently have non-specific and overlapping clinical and radiological features, and approximately 10–20% of patients with ILD are diagnosed with unclassifiable ILD [9]. Therefore, an accurate ILD diagnosis may sometimes require invasive procedures such as a surgical lung biopsy (SLB). However, SLB is associated with a substantial risk of complications [10], and thus, an alternative technique to evaluate cellular infiltration is warranted in the management of patients with ILD.

Radiomics involves converting medical images into high-dimensional data that reflect the underlying pathophysiology [11]. Radiomics features can effectively predict histologic subtypes of lung cancers [12, 13]. Moreover, some radiomics features have already been used for segmenting ILD lesions on computed tomography (CT) images [14]. Some reports have evaluated radiomics features in the patients with ILD [15, 16]. However, a direct comparison between CT radiomics features and histological specimens in the ILD patients has not been thoroughly performed. We hypothesized that CT radiomics features are related to cellular infiltration in SLB specimens of the ILD patients. Thus, this study aimed to create radiomics models to quantitatively and qualitatively predict the degree of cellularity based on the direct comparison of the CT radiomics features with the histology of fibrotic ILD.

## Materials and methods

### Study design and patients

This retrospective, single-center study was approved by the Institutional Review Board (Ethics Committee number KCRC-21-0037). The requirement for informed consent was waived owing to the retrospective nature of the study.

**Fig. 1:**
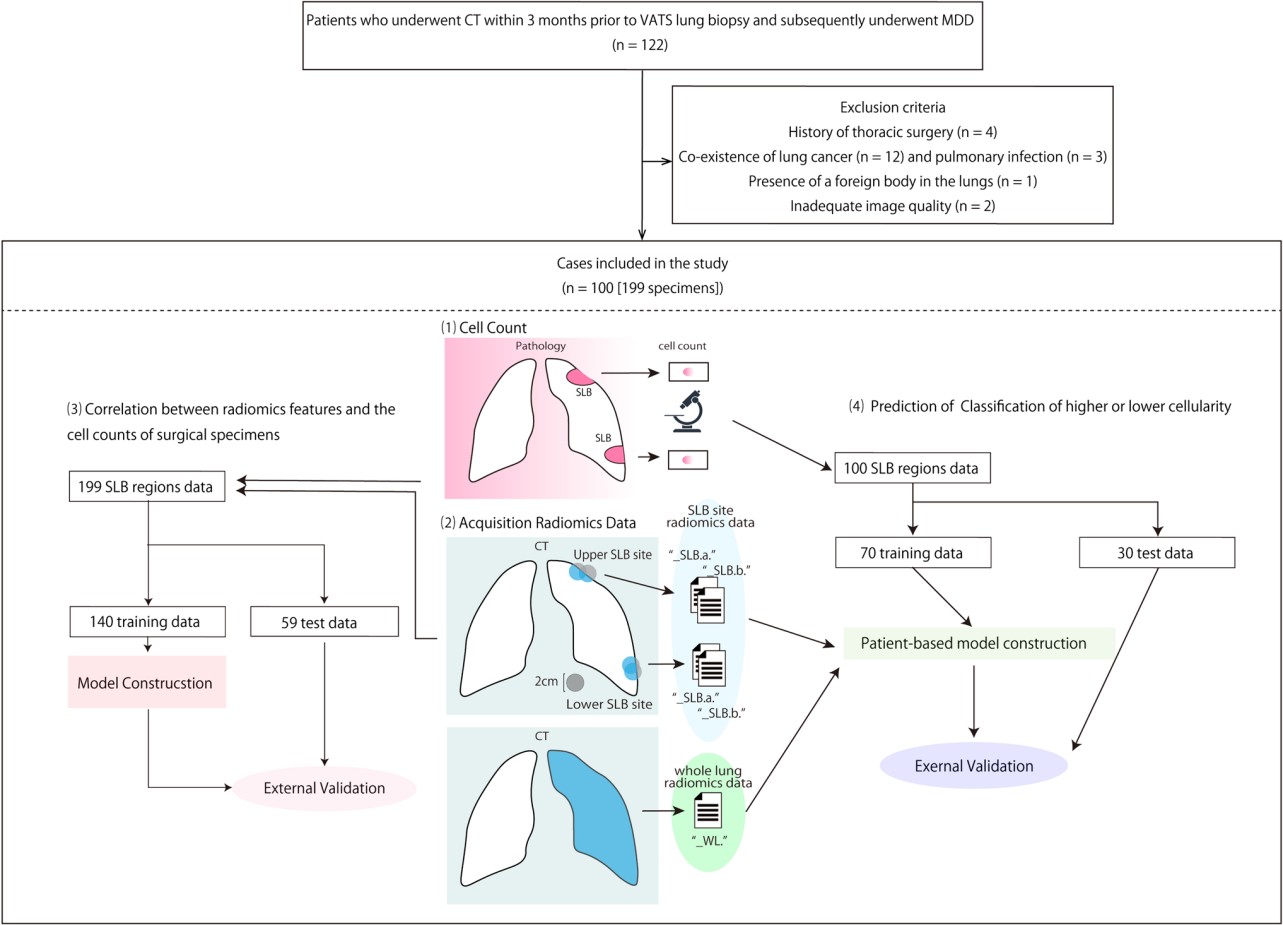
Study flowchart. SLB, video-assisted thoracic surgery; SLB, surgical lung biopsy

Patients with ILD who underwent thoracoscopic SLB between October 2018 and January 2022 were eligible (Fig. 1). We included 122 consecutive patients with fibrosing ILD who underwent CT examinations within 6 months before surgery. Patients with lung cancer (*n* = 12) and pulmonary infection (*n* = 3) were excluded, because these involve neoplastic cell proliferations. The patients who previously underwent thoracic surgery (*n* = 4), one with a foreign body in the lung (*n* = 1) and two with inadequate CT image quality (*n* = 2), were also excluded because of the potential undesirable artifacts. Baseline clinical measurements, including pulmonary function tests, were obtained within 3 months of the CT examinations. All baseline CT images were non-contrast, obtained using a thin-slice CT scanner (Aquilion Precision, Canon Medical Systems, Otawara, Japan) at full inspiration in the supine position with a tube voltage of 120 kVp and automatic tube current modulation. The median effective dose (interquartile range) of the CT scans was 8.23 (7.24–9.1) mSv. These data were calculated based on the dose–length products (mGy · cm) and a k-factor of 0.0140 (mSv · mGy^−1^ · cm^−1^). We used the CT images reconstructed using filtered-back projection and soft kernel, with 512 × 512 matrices and 0.5 mm slices. We automatically measured the total lung volume and the extent of each lesion using artificial intelligence-based software [Quantification by Ziosoft Informatics Platform for Interstitial Lung Disease (QZIP-ILD), Ziosoft, Tokyo, Japan] [17]. Two board-certified chest radiologists performed IPF and HP pattern classification according to the American Thoracic Society guidelines [1, 4] without clinical and pathological information. The findings were agreed upon in a consensus between the two radiologists.

### Biopsy and histopathological/morphometrical analysis

One pathologist with 20 years of experience in ILD selected a representative hematoxylin–eosin-stained slide for each SLB site. The slides were scanned using a virtual slide scanner (NanoZoomer, Hamamatsu Photonics, Hamamatsu, Japan). Then, we counted the number of nucleated cells and measured the tissue area using public morphometric software (QuPath v0.2.3) [18]. We calculated the number of nucleated cells per unit area (1000 μm^2^). Among specimens from the 99 patients with two SLB sites, those with the higher cell count were used for the analysis. We compared the cell counts between patients treated with and without anti-inflammatory therapy. A multidisciplinary discussion (MDD) that included specialists in respiratory medicine, diagnostic radiology, and pathology determined whether anti-inflammatory therapy was indicated. Based on a comprehensive review of the patient's clinical course, encompassing a detailed chronological medical history, thorough physical examination, and a series of laboratory and imaging tests subsequent to the initial diagnosis of MDD, the diagnosis of unclassifiable disease was revised during the departmental conference in respiratory medicine.

### Radiomics analysis and radiomics-based models

A schematic diagram of the region-of-interest (ROI) placement and construction of the specimen-based model and patient-based model is shown in Fig. 1. We positioned the ROI using surgical staples as a guide to indicate the biopsy site. The staple line measured 2–4 cm in length in the post-operative CT images. Therefore, we placed two volumes of interest (VOIs) to cover the staple line. Moreover, we measured the radiomics features. All VOIs were placed by an expert radiologist with 29 years of experience or a pneumologist with 18 years of experience. The center points of the two spherical VOIs with 2 cm diameter were manually placed to include a maximum volume of the interstitial lesion, as well as the portion removed by SLB in each patient. In usual interstitial pneumonia (UIP) pattern fibrosis, the lesion existed just below the pleura. Therefore, VOIs included some extrapulmonary regions. When the sphere contained extrapulmonary regions, these regions were automatically removed using QZIP-ILD [17]. All radiomics features were calculated using in-house software based on PyRadiomics (version 3.0.1; https://pyradiomics.readthedocs.io/en/latest/). In total, 107 radiomics features from the category of the histogram and gray-level co-occurrence matrix were extracted from each VOI and the whole lung.

The radiomics features obtained from the two VOIs placed for each SLB site were labeled as “SLB.a” and “SLB.b.” The order of labeling was arbitrary. Radiomics features extracted from regions obtained from whole lungs were labeled “_WL.” We built two radiomics-based models. One model predicts cell counts in the histological specimens (specimen-based model) with “SLB.a” and “SLB.b.”-labeled radiomics features, and another one predicts patients with abundant cellular infiltration in their histology specimens (patient-based model) with “SLB.a”, “SLB.b.” and “WL.”-labeled radiomics features.

#### Step-1: relationship of specimen-based model and histological cell count

To build the specimen-based model, 199 VOIs were divided into two datasets: training (140 specimens) and validation (59 specimens). A linear regression model with the least absolute shrinkage and selection operator (LASSO) [19] was applied to simultaneously construct a support vector machine (SVM) to predict the number of cells per unit area in surgical specimens and select significant radiomics features in the training dataset. A discordant pathological diagnosis between different biopsy sites is frequently observed in the same patient [20]. Therefore, we treated the specimens obtained from different biopsy sites in one patient as different samples.

#### Step-2: relationship of patient-based model and the dichotomized classification of cellularity

In clinical settings, the level of cellularity, whether high or low, is one of the crucial factors in justifying the indication for an anti-inflammatory therapy. Therefore, the patient-based model to classify the patient to high or low cellularity was developed. We divided patients into two groups, one with high cell counts and the other one with low cell counts. The median value of the histological cell counts across all patients was used to define the cut-off value to dichotomize the degree of cellularity: 50 high-cellularity cases and 50 low-cellularity cases. Seventy of these 100 cases were randomly selected and used as training datasets to build the model. Thirty separate cases were used as test data for external validation. We constructed this model using SVM with LASSO, the same as for the specimen-based model in Step 1.

### Statistical analysis

Continuous patient characteristics are summarized using descriptive statistics (n, mean, median, standard deviation, and interquartile range), while categorical patient characteristics are summarized using frequency (percentage). The histological cell counts were compared between the two groups, i.e., the anti-inflammatory therapy group and the non-anti-inflammatory therapy group, using Student’s t test.

In the specimen-based prediction model, for the evaluation of the relationship between the actual and predicted number of cells, the estimated model was evaluated using a root-mean-square error in the validation dataset, and the correlation coefficient (R) and *p* value of the linear fit were calculated. The R package ‘glmnet’ (https://cran.r-project.org/) was used for the model construction.

In the patient-based prediction model, the median cell count was used as a cut-off to classify patients into two groups, and the model was constructed using the R package ‘glmnet’. The model’s performance was evaluated by the F1 score and the accuracy of the result of validation.

To compare the radiomics features between the specimen-based and patient-based models, the top five features in regression coefficient value were selected for each of the specimen-based model and patient-based model in the order of absolute value, and a heat map was created to show the correlation between them.

## Results

### Patient characteristics

A total of 100 patients (61 men and 39 women; mean age, 62 ± 8.9 years) were included in this study (Fig. 1). None of the patients had received treatment with steroids or other immunosuppressants. Table 1 shows the patient characteristics, including respiratory function test and MDD results. Table 2 shows the CT pattern classification according to the American Thoracic Society guidelines [1, 4] and ILD volume analysis using Quantification by Ziosoft Informatics Platform for Interstitial Lung Disease. Based on the visual interpretation by the radiologists, 66 patients had indeterminate UIP, 2 showed probable UIP patterns, and 32 showed alternative diagnoses. In all the cases of alternative diagnoses, fibrosis was observed in histological findings. After MDD, the diagnosis of these patients was established as CTD (*n* = 16), HP (*n* = 27), IPF (*n* = 19), unclassifiable ILD (*n* = 32) that included two cases that met IPAF criteria, idiopathic non-specific interstitial pneumonia (*n* = 5), and desquamative interstitial pneumonia (*n* = 1). Two patients initially determined to have unclassifiable disease in the MDD were later diagnosed with connective tissue diseases, including rheumatoid arthritis and systemic scleroderma, during follow-up. Similarly, one patient was later identified as having fibrotic HP. Among these 100 patients, 45 were treated with anti-inflammatory therapy according to the MDD.

**Table 1:** Patient characteristics

Characteristic	*N* = 100
Demographics	
Sex, *n* (%)	
Female	39 (39)
Male	61 (61)
Smoking history, *n* (%)	
Ever	66 (66)
Never	34 (34)
Smoking index, median (IQR)	205 (0–760)
Age (years), median (IQR)	64 (57–68)
Days from CT to SLB (days), median (IQR)	7 (5–14)
Respiratory function test, mean ± SD	
%DL_CO_,	77 ± 18
%DLCO/VA	94 ± 22
%FEV_1.0_	86 ± 15
%FVC	96 ± 71
%TLC	86 ± 17
%VC	85 ± 17
Decisions at MDD	
Diagnosis, *n* (%)	
CTD or IPAF	31 (31)
DIP	1 (1.0)
fNSIP	4 (4.0)
HP	27 (27)
IPF	19 (19)
Unclassified	18 (18)
Indication for anti-inflammatory therapy, *n* (%)	
Yes	45 (45)
No	55 (55)
Indication for anti-inflammatory therapy or HP work-up, n (%)	66 (66)

*IQR* interquartile range, *SLB* surgical lung biopsy, *DLCO* diffusing capacity of the lungs for carbon monoxide, *SD* standard deviation, *VA* alveolar volume, *FEV1.0* forced expiratory volume in 1 s, *FVC* forced vital capacity, *TLC* total lung capacity, *VC* vital capacity, *MDD* multidisciplinary discussion, *CTD* connective tissue disease, *IPAF* interstitial pneumonia with autoimmune features, *DIP* desquamative interstitial pneumonia, *fNSIP* fibrotic non-specific interstitial pneumonia, *HP* hypersensitivity pneumonitis, *IPF* idiopathic pulmonary fibrosis, *UIP* usual interstitial pneumonia

**Table 2:** CT characteristics

CT characteristic	*N* = 100
CT pattern classification in ATS guidelines	
IPF pattern, *n* (%)	
UIP	0 (0)
Probable	2 (2.0)
Indeterminate	66 (66)
Alternative	32 (32)
HP pattern, *n* (%)	
Typical	1 (1.0)
Compatible	43 (43)
Indeterminate	56 (56)
QZIP-ILD analysis, median (IQR)	
All lung volume (mL)	3,662 (3147–4419)
Emphysema (%)	0.35 (0.16–1.03)
Consolidation (%)	1.23 (0.83–2.13)
Consolidation with traction bronchiectasis (%)	0.88 (0.51–1.46)
GGO (%)	10 (5–15)
Honeycomb (%)	0.03 (0.01–0.11)
Reticulation (%)	6.2 (3.4–8.8)
Traction bronchiectasis (%)	0.97 (0.58–1.53)
Normal (%)	78 (70–86)

*IQR* interquartile range, *HP*, hypersensitivity pneumonitis, *IPF* idiopathic pulmonary fibrosis, *ATS* American thoracic society, *UIP* usual interstitial pneumonia, *QZIP-ILD*  quantification by ziosoft informatics platform for interstitial lung disease, *GGO* ground-glass opacity

### Cell number in SLB and anti-inflammatory therapy

**Fig. 2:**
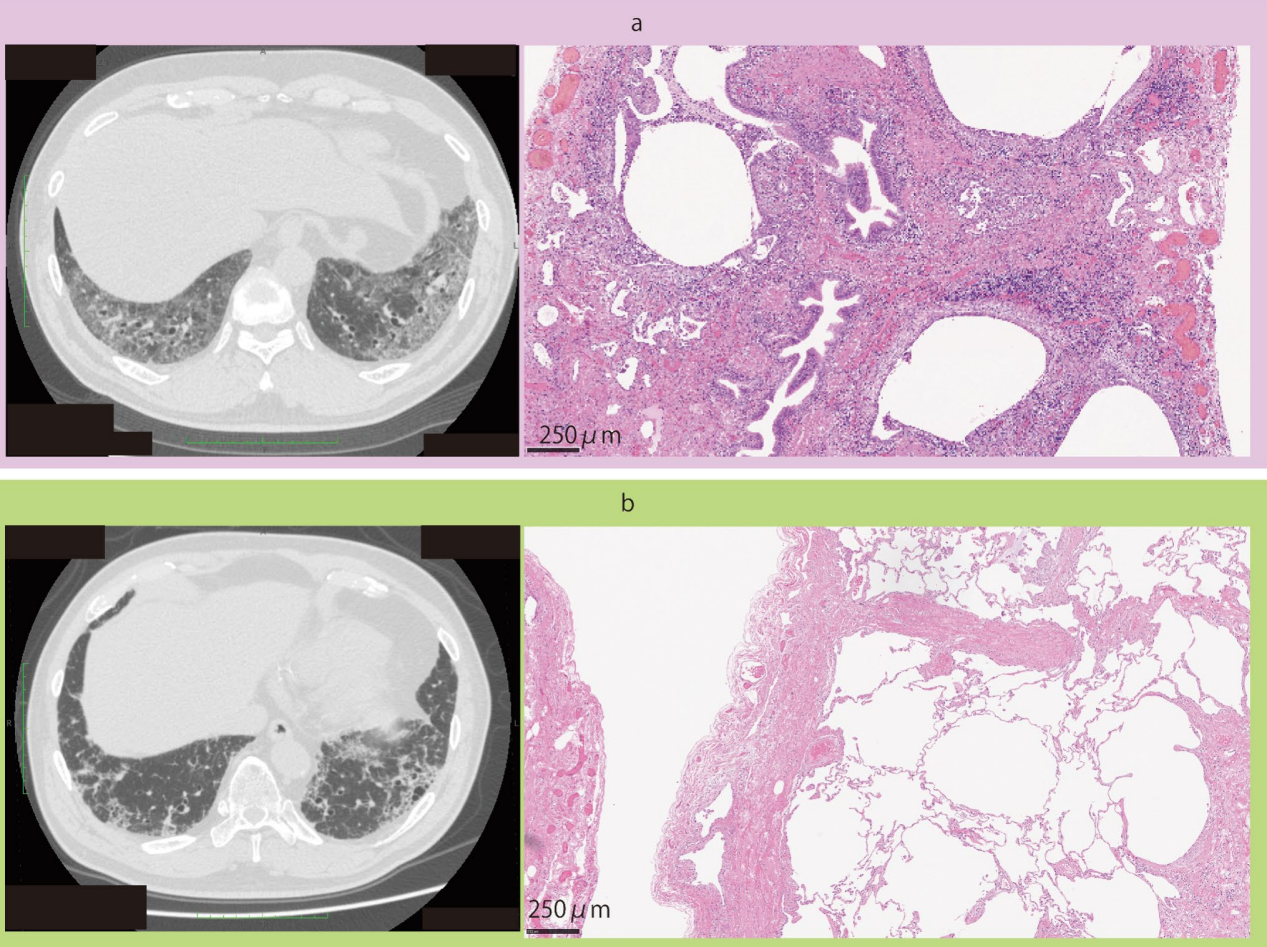
CT and histological slides of representative cases. (**a**) A 68-year-old man with high cell counts per unit area (4.32 cells/1000 μm^2^) necessitating anti-inflammatory therapy and is classified by the model as belonging to the high-cellularity group. (**b**) A 63-year-old man with low cell counts per unit area (2.32 cells/1000 μm^2^), for whom anti-inflammatory therapy is not indicated, who is classified by the model into the low-cellularity group

**Fig. 3:**
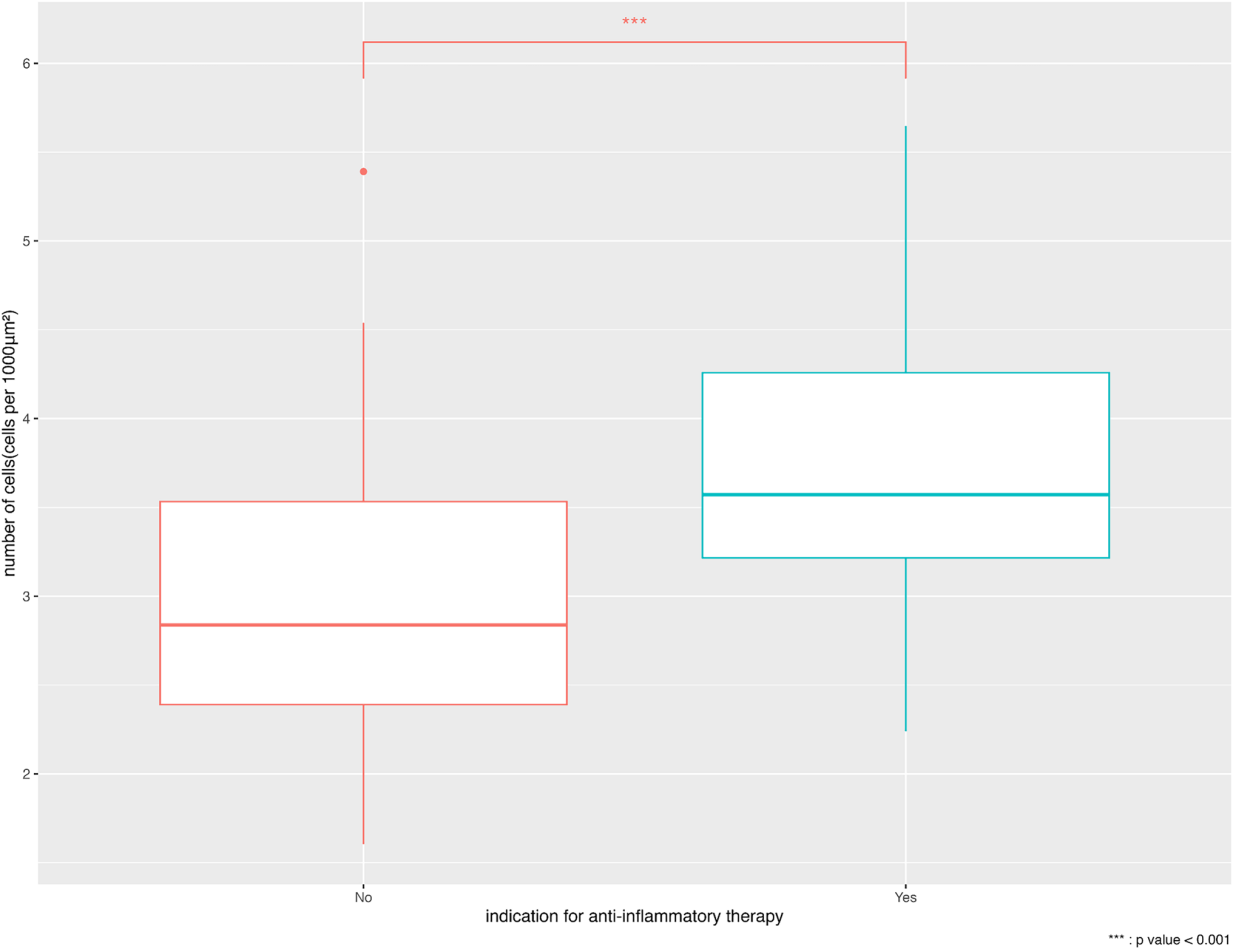
Box-and-whisker plot of cell counts for the groups according to eligibility for anti-inflammatory therapy in the multidisciplinary discussion

Ninety-nine patients had two SLB sites (upper and lower), and 1 had only one SLB site due to adhesions. The median and interquartile range of the number of cells were 2.5/1000 μm^2^ (2.16–3.06) and 3.1/1000 μm^2^ (2.39–3.79) in the upper and lower SLB sites, respectively. Finally, 199 pathological specimens from 100 patients were included in the study. Figure 2 shows two representative cases: a patient with unclassifiable ILD with a large amount of cellular infiltration (Fig. 2a) and a patient with IPF showing peri-lobular fibrosis without marked cellular infiltration (Fig. 2b). The cell count in the specimens of the patients with indication for steroid treatment in the MDD was significantly greater than that in the patients without indications for anti-inflammatory therapy (*p* < 0.001) (Fig. 3).

#### Step-1: relationship of specimen-based model with histological cell count

**Fig. 4:**
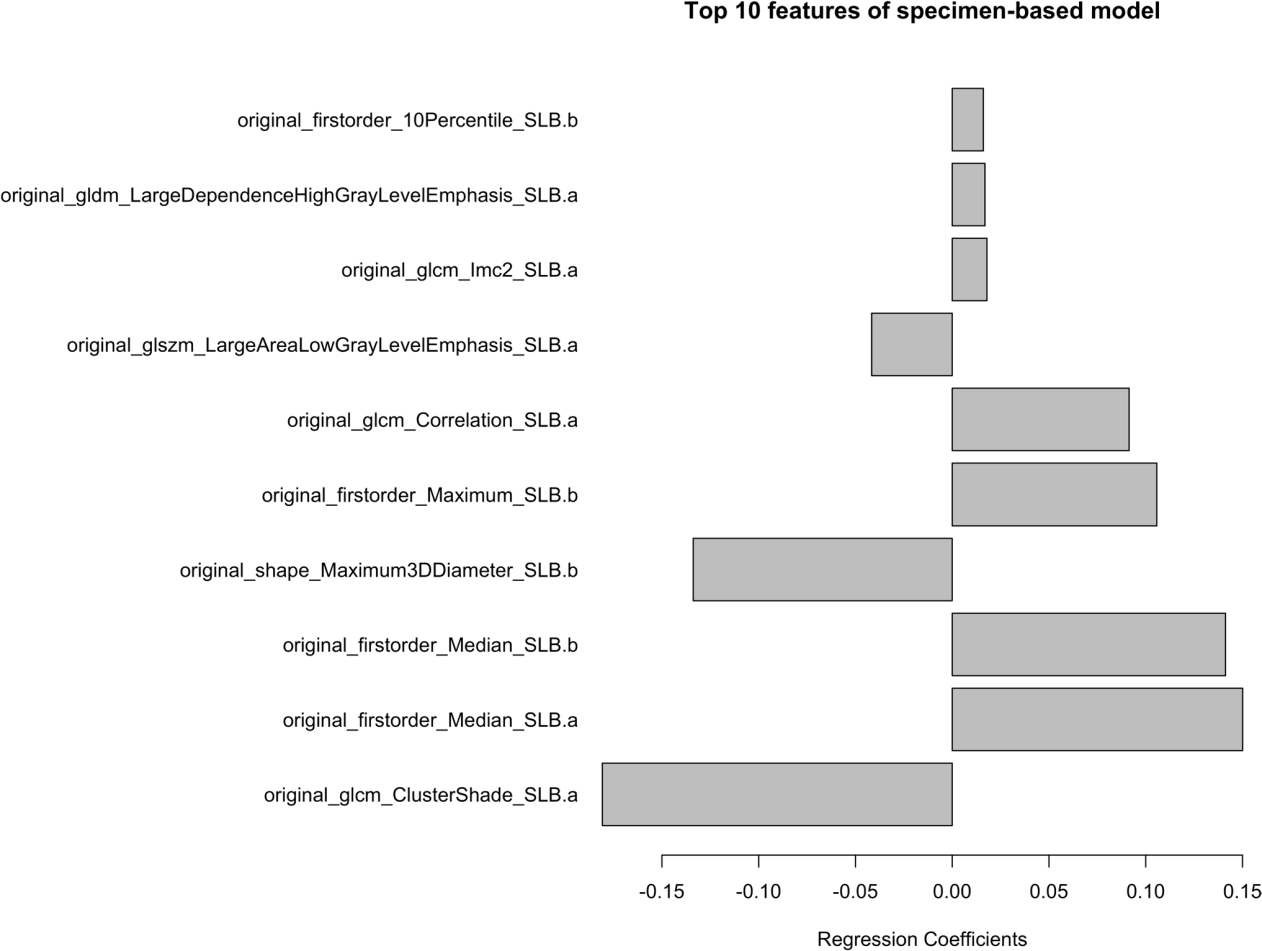
The top 10 radiomics features of the specimen-based model. gldm, Gray Level Dependence Matrix; glcm, Gray Level Co-occurrence Matrix; IMC, Informational measure of correlation; glszm, Gray Level Size Zone Matrix; gldm, Gray Level Dependence Matrix

**Fig. 5:**
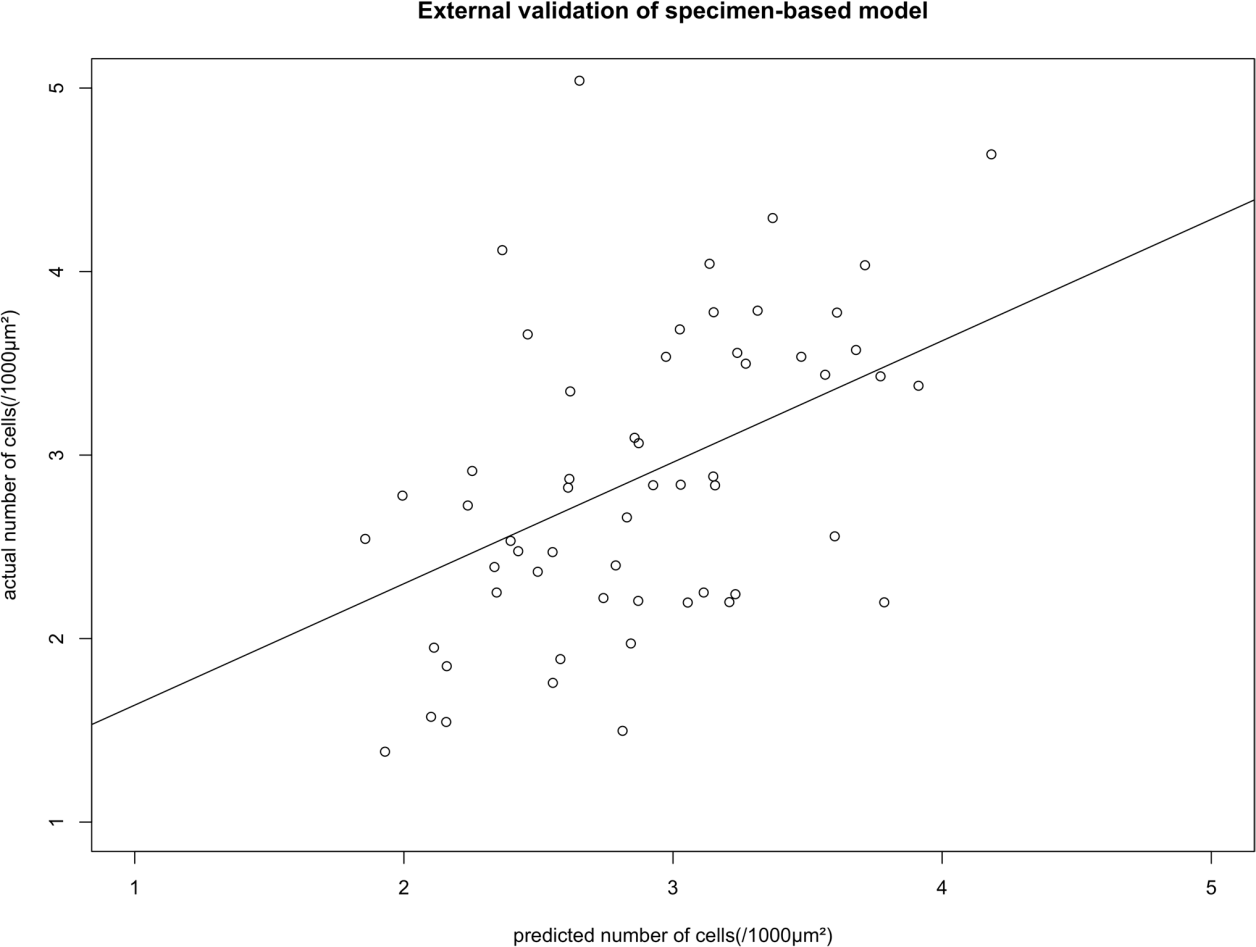
External validation of the specimen-based radiomics model. The horizontal axis is the number of cells predicted by the model, and the vertical axis is the actual number of cells. SLB, surgical lung biopsy

We constructed a model to predict cell count in the specimens. In the training dataset, 14 radiomics features were selected (Supplementary Table 1). Figure 4 shows the top 10 radiomics features used in this model. The most significant coefficient was “original_glcm_ClusterShade_SLB.a,” followed by “original_firstorder_Median_SLB.a.” Fig. 5 shows the correlation between the predicted cell count using this radiomics model and the actual cell count measured in the external validation set consisting of 59 specimens. The root-mean-square error was 0.797. The *p* value between predicted and measured values was less than 0.001, and the correlation coefficient was 0.48.

#### Step-2: relationship of patient-based model with the dichotomized classification of cellularity

**Fig. 6:**
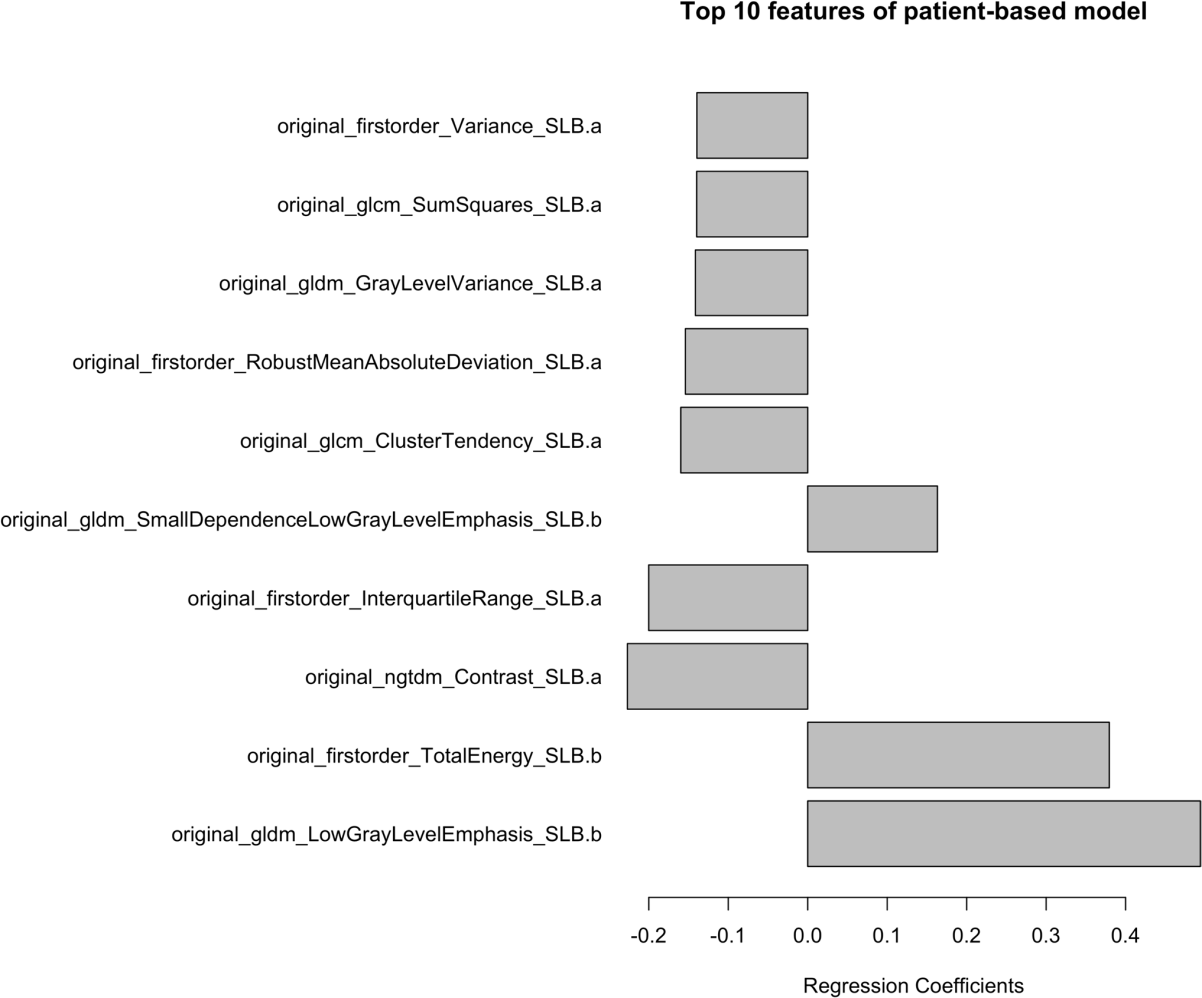
Top 10 radiomics features of the patient-based model. glcm, Gray Level Co-occurrence Matrix; gldm, Gray Level Dependence Matrix; ngtdm, Neighboring Gray Tone Difference Matrix

The median number of cells in the patient was 3.27 (/1000 μm^2^). The patients were divided into two groups, 50 patients in each, one with the higher cell count and the other one with lower cell count. We created a patient-based model predicting higher or lower cellularity using 70 training sets chosen randomly from all sets. Supplementary Table 2 shows patient backgrounds in the training and test dataset. The training dataset had a significantly higher percentage of DL_CO_, but there was no difference in other patient backgrounds including quantitative CT results by QZIP-ILD. As a result, 22 radiomics features were selected to predict patients with higher cellularity (Supplementary Table 3). The top 10 selected radiomics features are shown in Fig. 6. The performance of this model was evaluated using external validation. The accuracy was 70.0%, F1 score 0.73, and the area under the ROC curve 0.70.

### Radiomics features’ correlation

Some radiomics features in these models were correlated with each other. Specifically, “original_firstorder_Median_SLB.a” and “original_firstorder_Median_SLB.b” were negatively correlated with “original_gldm_LowGrayLevelEmphasis_SLB.b”, “original_firstorder_TotalEnergy_SLB.b” and “original_gldm_SmallDependenceLowGrayLevelEmphasis_SLB.b”, and positively correlated with “original_firstorder_InterquartileRange_SLB.a” (Supplementary Fig. 1).

## Discussion

This study directly compared the cell count in histological specimens obtained using surgical biopsy with the CT radiomics features of the biopsy sites in patients with ILD. We constructed a CT radiomics model to predict the cell count in 140 histological specimens used as training datasets. This model predicted the actual cell count measured in 59 specimens used for external validation (root-mean-square error: 0.797). Given that the median cell count per 1000 μm^2^ was 2.5 in the group without indication for anti-inflammatory therapy and 3.1 in that with indication, these numbers are not sufficient to determine a treatment strategy on their own. However, it is suggested that radiomics can predict cell counts, albeit with insufficient accuracy in this particular case. It is expected that the accuracy will increase with the increase in the number of n and number of facilities in the future and that the method will become more practical. The CT radiomics model, trained using the biopsy site and whole lung data, to predict higher cellular infiltration showed good performance, with F1 score of 0.73 in the external validation. To the best of our knowledge, this is the first study to compare CT radiomics features with histological specimens directly in patients with ILD.

In this study, approximately two-thirds of the patients showed an “indeterminate for UIP” CT pattern; these patients could have had either IPF, HP, CTD, or unclassifiable ILDs [21]. In these situations, histological findings may support the diagnosis, e.g., granuloma and giant cells may suggest HP [22, 23], and the presence of inflammatory cellular infiltration may support CTD or IPAF [3]. Mäkelä et al. reported that high numbers of interstitial mononuclear inflammatory cells and intra-alveolar macrophages were associated with prolonged survival in patients with IPF in the Finnish IPF registry [24]. Treatment has not been completely established for these unclassifiable ILDs. However, Yamano et al. reported that immunosuppressive therapy provided better survival for the patients with IPAF whose histological findings were similar to UIP and inflammatory cellular infiltration than anti-fibrotic treatment [5].” These studies support the importance of inflammatory cellular infiltration in patients with fibrotic ILD. Our results showed that CT radiomics could predict the degree of inflammatory cell infiltration, and we posit that CT radiomics has the potential to help clinicians choose treatment strategies for ILDs.

In the specimen-based model, using CT radiomics features at the biopsy sites, original_glcm_ClusterShade_SLB.a, original_firstorder_Median_SLB.a, original_firstorder_Median_SLB.b, and other features were selected. Original_firstorder_Median directly reflects the CT attenuation values. Higher cellularity will likely increase CT attenuation values in the lesion. Our result is consistent with that of a previous report that showed a positive correlation between the cellularity of the surgical specimens and CT attenuation values at the biopsy site in patients with ILDs [25]. Original_glcm_ClusterShade was one of the features that showed a sharp boundary of the lesion, which tended to be slightly lower in SLB sites with higher cellularity. The typical histological feature of UIP encompasses spatial heterogeneity, where normal lung tissue exists next to peri-lobular fibrosis with a sharp boundary, and cellular infiltration is low, culminating in elevated levels of Original_glcm_ClusterShade [23]. Conversely, it was postulated that regions exhibiting higher cellularity might display diminished demarcation, consequently yielding reduced levels of Original_glcm_ClusterShade.

Meanwhile, non-specific interstitial pneumonia commonly seen in ILD with higher inflammatory cellularity shows a uniform lesion distribution, and the boundary between fibrosis and normal lung tissue is ill defined [26]. In the patient-based model, the top 10 selected features were from SLB. The most significant feature selected was original_gldm_LowGrayLevelEmphasis_SLB.b. This feature exhibited higher values when low CT values were uniformly distributed throughout the area. Ground-glass opacities, commonly seen in lesions with substantial cellular infiltration, are well known. Therefore, it is plausible that this imaging characteristic effectively captures the essence of such lesions, indicating a homogenous presence of cellular infiltrates as reflected in uniformly low CT values.

After creating the models, we needed to analyze the correlation of each of the automatically selected radiomics features in each model, because it was necessary to examine why different radiomics features were selected. Most of the features were different between the specimen-based and patient-based models. These radiomics features were correlated with each other, and some degree of correlation was observed when the influential features of the two models were compared (Supplementary Fig. 1). Specifically, “original_firstorder_Median_SLB (.a and.b)” and “original_firstorder_TotalEnergy_SLB.b” were strongly negatively correlated with each other. These correlated features may have a similar role, which explains the inconsistent feature selection.

This study had some limitations. First, this was a retrospective study performed in a single center, and we used CT images obtained with one type of CT scanner, which limited the generalizability of our results. Although cohort reliability is questionable due to the bias from being a single site, it was difficult to calibrate, because we used SVM to build the predictive model. Other than a significant difference in DL_co_, there were no differences between the training and test datasets. Future studies should include multicenter studies and predictive models that allow for calibration to show sufficient reliability for the cohort. Second, the patients with high inflammatory cell infiltration were grouped together in our study. It should be more practical to develop a model that detects cases with a UIP pattern fibrosis but with a severe lymphocytic infiltration. However, it was difficult to create such a model owing to the small sample size. In addition, SLB was performed in our study population for the diagnosis of ILD, because non-invasive imaging diagnosis was difficult, and the initial treatment was not effective enough even in the patients who were finally confirmed as HP and DIP. Therefore, our radiomics model could be biased toward the patients in the critical or complicated situation. In the patients with typical HP and DIP, antigen avoidance for HP and smoking cessation for DIP are the basic first treatment strategy, but anti-inflammatory therapy may be a treatment option in a case with high inflammatory cell infiltration, and we believe that our model might potentially provide a useful information in HP and DIP. Moreover, our study's limited population size and technical limitations precluded the development of a more sophisticated model, such as one involving optimal subset causality regression [27]. This is in contrast with Schniering et al., who employed CT radiomics to analyze scleroderma-associated ILDs in a dataset of 156 samples with 1386 features [15]. Furthermore, the cut-off values for the two categories in the patient-based model might have been better had they been set to optimize the more clinically important criteria, such as the indication for anti-inflammatory treatment. However, owing to the low number of cases, a biased number of cases in the test data for the two categories caused by an off-median cut-off would have prevented a predictive model from being created. Third, our model could not distinguish between high lymphocytic and high macrophage infiltration. Further studies should be conducted for more detailed evaluation of lung inflammation. Despite this, the significant differences observed in the indications for anti-inflammatory treatment when total nucleated cells were evaluated indicate that our analysis method is still valid. Finally, our study was a pilot study; a prospective, multicenter study with a larger population is necessary to overcome the study limitations.

In conclusion, our direct comparison of CT radiomics features with histological specimens from SLB enabled the development of predictive models to assess cellular infiltration in ILD. The radiomics features identified provide valuable insights for both quantitative and qualitative evaluations of cellular infiltration severity in ILD. Leveraging these features in quantitative CT analysis could potentially compensate for the limitations of visual CT interpretation and SLB.

## Supplementary Information

Below is the link to the electronic supplementary material.

Supplementary file1 Supplementary Fig.1 Correlation matrix of top 5 radiomics features of specimen-based model and patient-based model. glcm, Gray Level Co-occurrence Matrix; gldm, Gray Level Dependence Matrix; ngtdm, Neighboring Gray Tone Difference Matrix (TIFF 91317 KB)

Supplementary file2 (DOCX 25 KB)
