# Supplementary material for: Correlation of CT-based radiomics analysis with pathological cellular infiltration in fibrosing interstitial lung diseases
Source: Jpn J Radiol. 2024 Jun 18;42(10):1157–67. doi: 10.1007/s11604-024-01607-2 (PMC11442537; doi:10.1007/s11604-024-01607-2)
Supplement: Supplementary file 2 — Supplementary file2 (DOCX 25 KB) [file 11604_2024_1607_MOESM2_ESM.docx]

**Supplemental Table 1: Selected Radiomics Features in the Specimen-based Model (Step-1)**

| Radiomics Features | **Coefficient** |
| --- | --- |
| original_glcm_ClusterShade_SLB.a | -0.1807974 |
| original_firstorder_Median_SLB.a | 0.1500840 |
| original_firstorder_Median_SLB.b | 0.1412129 |
| original_shape_Maximum3DDiameter_SLB.b | -0.1338530 |
| original_firstorder_Maximum_SLB.b | 0.1057148 |
| original_glcm_Correlation_SLB.a | 0.0914199 |
| original_glszm_LargeAreaLowGrayLevelEmphasis_SLB.a | -0.0416262 |
| original_glcm_IMC2_SLB.a | 0.0179644 |
| original_gldm_LargeDependenceHighGrayLevelEmphasis_SLB.a | 0.0169165 |
| original_firstorder_10Percentile_SLB.b | 0.0160836 |
| original_shape_MajorAxisLength_SLB.a | 0.0128967 |
| original_firstorder_Minimum_SLB.a | 0.0116942 |
| original_glszm_ZoneVariance_SLB.a | -0.0001017 |

glcm, Gray Level Co-occurrence Matrix; SLB, surgical lung biopsy; glszm, Gray Level Size Zone Matrix; gldm, Gray Level Dependence Matrix; IMC, Informational Measure of Correlation, gldm; gray level dependence matrix

**Supplemental Table 2: Comparison of clinical and CT features between patients in training and test groups**

| **Characteristic** | Training, N = 70 | Test, N = 30 | **p-value** |
| --- | --- | --- | --- |
| Male, n (%) |  |  | 0.75 |
| Female | 28 (40) | 11 (37) |  |
| Male | 42 (60) | 19 (63) |  |
| smoking history, n (%) |  |  | 0.93 |
| Ever | 46 (66) | 20 (67) |  |
| Never | 24 (34) | 10 (33) |  |
| smoking index, Median (IQR) | 305 (0 – 800) | 168 (0 – 694) | 0.54 |
| Age(yr), Median (IQR) | 65 (58 – 69) | 58 (56 – 67) | 0.069 |
| %DL_CO_, Mean ± SD | 80 ± 18 | 69 ± 16 | 0.010 |
| %DL_CO_/VA, Mean ± SD | 98 ± 21 | 87 ± 24 | 0.080 |
| %FEV_1.0_, Mean ± SD | 86 ± 15 | 86 ± 14 | 0.97 |
| %FVC, Mean ± SD | 90 ± 19 | 110 ± 127 | 0.74 |
| %TLC, Mean ± SD | 88 ± 18 | 81 ± 13 | 0.10 |
| %VC, Mean ± SD | 86 ± 18 | 83 ± 15 | 0.49 |
| Diagnosis, n (%) |  |  | 0.61 |
| CTD | 10 (14) | 6 (20) |  |
| DIP | 0 (0) | 1 (3.3) |  |
| fNSIP | 3 (4.3) | 2 (6.7) |  |
| HP | 19 (27) | 8 (27) |  |
| IPF | 15 (21) | 4 (13) |  |
| Unclassified | 23 (33) | 9 (30) |  |
| indication for anti-inflammatory therapy, n (%) | 30 (43) | 15 (50) | 0.51 |
| indication for anti-inflammatory therapy or HP work-up, n (%) | 44 (63) | 22 (73) | 0.31 |
| Days from CT to SLB (days), Median (IQR) | 7 (5 – 12) | 10 (6 – 20) | 0.14 |
| IPF pattern, n (%) |  |  | 0.47 |
| alternative | 21 (30) | 11 (37) |  |
| indeterminate | 48 (69) | 18 (60) |  |
| probable | 1 (1.4) | 1 (3.3) |  |
| HP pattern, n (%) |  |  | 0.17 |
| compatible | 33 (47) | 10 (33) |  |
| indeterminate | 37 (53) | 19 (63) |  |
| typical | 0 (0) | 1 (3.3) |  |
| All lung volume(ml), Median (IQR) | 3,695 (3,154 – 4,737) | 3,644 (3,063 – 4,183) | 0.39 |
| Emphysema(%), Median (IQR) | 0.39 (0.18 – 0.84) | 0.28 (0.09 – 2.41) | 0.66 |
| Consolidation(%), Median (IQR) | 1.16 (0.72 – 1.72) | 1.37 (1.07 – 2.19) | 0.14 |
| Consolidation with traction bronchiectasis(%), Median (IQR) | 0.84 (0.49 – 1.40) | 0.97 (0.57 – 1.47) | 0.89 |
| GGO(%), Median (IQR) | 9 (5 – 15) | 11 (7 – 17) | 0.080 |
| Honey comb(%), Median (IQR) | 0.04 (0.00 – 0.16) | 0.03 (0.01 – 0.08) | 0.77 |
| Reticulation(%), Median (IQR) | 5.6 (3.2 – 8.5) | 7.0 (3.9 – 9.9) | 0.22 |
| Traction bronchiectasis(%), Median (IQR) | 0.89 (0.57 – 1.47) | 1.05 (0.59 – 1.77) | 0.45 |
| Normal(%), Median (IQR) | 79 (71 – 86) | 75 (64 – 83) | 0.10 |

IQR, interquartile range; SLB, surgical lung biopsy; DLCO, diffusing capacity of the lungs for carbon monoxide; SD, standard deviation; VA, alveolar volume; FEV1.0, forced expiratory volume in 1 second; FVC, forced vital capacity; TLC, total lung capacity; VC, vital capacity; MDD, multidisciplinary discussion; CTD, connective tissue disease; IPAF, interstitial pneumonia with autoimmune features; DIP, desquamative interstitial pneumonia; fNSIP, fibrotic non-specific interstitial pneumonia; HP, hypersensitivity pneumonitis; IPF, idiopathic pulmonary fibrosis; UIP, usual interstitial pneumonia

**Supplemental Table 3: Selected Radiomics Features in the Patient-based Model (Step-2)**

| Radiomics Features | Coefficients |
| --- | --- |
| original_gldm_LowGrayLevelEmphasis_SLB.b | 0.4940868 |
| original_firstorder_TotalEnergy_SLB.b | 0.3794876 |
| original_ngtdm_Contrast_SLB.a | -0.2269366 |
| original_firstorder_InterquartileRange_SLB.a | -0.2001137 |
| original_gldm_SmallDependenceLowGrayLevelEmphasis_SLB.b | 0.1632261 |
| original_glcm_ClusterTendency_SLB.a | -0.1597617 |
| original_firstorder_RobustMeanAbsoluteDeviation_SLB.a | -0.1539878 |
| original_gldm_GrayLevelVariance_SLB.a | -0.1413861 |
| original_glcm_SumSquares_SLB.a | -0.1397210 |
| original_firstorder_Variance_SLB.a | -0.1394758 |
| original_shape_MajorAxisLength_SLB.a | -0.0941443 |
| original_shape_Elongation_SLB.a | 0.0933904 |
| original_glrlm_GrayLevelVariance_SLB.a | -0.0928299 |
| original_firstorder_MeanAbsoluteDeviation_SLB.a | -0.0872993 |
| original_firstorder_90Percentile_SLB.a | -0.0802668 |
| original_firstorder_RootMeanSquared_SLB.b | 0.0732653 |
| original_shape_Maximum2DDiameterSlice_SLB.a | 0.0690304 |
| original_firstorder_TotalEnergy_SLB.a | -0.0560927 |
| original_gldm_DependenceEntropy_SLB.a | -0.0422760 |
| original_glcm_Correlation_SLB.a | -0.0309304 |
| original_shape_Sphericity_WL | -0.0047947 |
| original_shape_Sphericity_SLB.a | -0.0013157 |

SLB, surgical lung biopsy; gldm, Gray Level Dependence Matrix; ngtdm, neighboring gray tone difference matrix; glcm, gray level co-occurrence matrix; gldm, gray level dependence matrix; glrlm, gray level run length matrix; WL, whole lung
